# The Reliability of the Wisconsin Card Sorting Test in Clinical Practice

**DOI:** 10.1177/1073191119866257

**Published:** 2019-08-02

**Authors:** Bruno Kopp, Florian Lange, Alexander Steinke

**Affiliations:** 1 Hannover Medical School, Hannover, Germany; 2 KU Leuven, Leuven, Belgium

**Keywords:** Wisconsin Card Sorting Test, reliability, split-half reliability, test-retest reliability, reliability generalization

## Abstract

The Wisconsin Card Sorting Test (WCST) represents the gold standard for the neuropsychological assessment of executive function. However, very little is known about its reliability. In the current study, 146 neurological inpatients received the Modified WCST (M-WCST). Four basic measures (number of correct sorts, categories, perseverative errors, set-loss errors) and their composites were evaluated for split-half reliability. The reliability estimates of the number of correct sorts, categories, and perseverative errors fell into the desirable range (*rel* ≥ .90). The study therefore disclosed sufficiently reliable M-WCST measures, fostering the application of this eminent psychological test to neuropsychological assessment. Our data also revealed that the M-WCST possesses substantially better psychometric properties than would be expected from previous studies of WCST test-retest reliabilities obtained from non-patient samples. Our study of split-half reliabilities from discretionary construed and from randomly built M-WCST splits exemplifies a novel approach to the psychometric foundation of neuropsychology.

The Wisconsin Card Sorting Test (WCST) was developed by Berg and Grant (Berg, 1948; Grant & Berg, 1948) to assess abstraction and the ability to shift cognitive strategies in response to changing environmental contingencies. The WCST is considered a measure of executive function (Heaton, Chelune, Talley, Kay, & Curtiss, 1993; Strauss, Sherman, & Spreen, 2006). It consists of four stimulus cards, placed in front of the subject: They depict a red triangle, two green stars, three yellow crosses, and four blue circles, respectively. The subject receives two sets of 64 response cards, which can be categorized according to color, shape, and number. The subject is told to match each of the response cards to one of the four stimulus cards and is given feedback on each trial whether he or she is right or wrong. The task requires establishing cognitive sets (e.g., to sort cards according to the abstract color dimension). This requirement of the task accounts for its ability to provide a measure of abstraction. Once a cognitive set has been established, it is necessary to maintain it in response to positive feedback. On the contrary, shifting the prevailing cognitive set is requested in response to negative feedback. The task provides information on several aspects of executive function beyond basic indices as task success or failure. Important indices of performance on the task include the number of categories achieved (i.e., the number of sequences of 6 or 10 consecutive correct sorts; the actual length of the sequence depends on the test version), the number of perseverative errors (i.e., the number of failures to shift cognitive set in response to negative feedback), and the number of set-loss errors (i.e., the number of failures to maintain cognitive set in response to positive feedback).

Although most practitioners use Heaton’s standard version (Heaton et al., 1993), there are other versions of the test. The standard WCST includes some ambiguous stimuli that can be sorted according to more than one category. Nelson (1976) removed all those response cards that shared more than one attribute with one of the stimulus cards in her Modified Card Sorting Test (MCST) such that it included only two sets of 24 non-ambiguous response cards. Schretlen (2010) more recently published the Modified Wisconsin Card Sorting Test (M-WCST), which represents a standardized version of Nelson’s (1976) MCST. An extensive review of the W/M/M-W//CST literature would be beyond the scope of this article; the interested reader is referred to previous publications (Demakis, 2003; Lange, Brückner, Knebel, Seer, & Kopp, 2018; Lange, Seer, & Kopp, 2017; Lezak, Howieson, Bigler, & Tranel, 2012; MacPherson, Sala, Cox, Girardi, & Iveson, 2015; Nyhus & Barceló, 2009; Strauss et al., 2006).

Many practitioners consider the WCST as the gold standard for the clinical assessment of executive function (see Rabin, Barr, & Burton, 2005, for a survey; Strauss et al., 2006). Nonetheless, information about a core psychometric property of the test, namely its reliability, is in surprisingly short supply. Reliability is a fundamental problem for measurement simply because it is difficult to accept misleading effects that fallible measurements might exert on decision making. Hence, psychologists have long studied the problem of reliability, in an attempt to understand how to estimate reliability as well as the ways to use these estimates (Cho, 2016; Nunnally & Bernstein, 1994; Revelle & Condon, 2018). At the heart of it is the issue that reliability indicators are needed for estimating confidence intervals around any measurement (Charter, 2003; Slick, 2006). Therefore, information about the reliability of any measure is fundamental not just for psychometricians but also for scientists and practitioners, who typically conduct neuropsychological assessment in the context of important decisions being made about individuals. An influential psychometric textbook recommended that “a reliability of .90 is the bare minimum, and a reliability of .95 should be considered the desirable standard” (Nunnally & Bernstein, 1994, p. 265).

Few studies examined the reliability of W/MCST scores. Their results suggest that the W/MCST scores are far from reaching the desirable standard of reliability. Lineweaver, Bondi, Thomas, and Salmon (1999) provided the study with the largest sample size, and their results can be regarded as being quite representative for the field as a whole (see Kopp, Maldonado, Hendel, & Lange, 2019; see also Appendix A Table S1 in the Supplemental Appendix, available online for an overview). Based on test-retest data from 142 healthy volunteers, these authors computed reliability estimates for MCST scores as low as .56 (number of categories), .64 (number of perseverative errors), and .46 (number of non-perseverative errors, which roughly corresponds to number of set-loss errors). Such low estimates of W/MCST score reliability raise the issue whether or not W/MCST scores could be confidently utilized in clinical practice, thereby putting the legitimacy of the W/MCST as a means to quantitatively assess abilities in executive function into doubt (Bowden et al., 1998).

Our present study was concerned with estimating M-WCST reliability as applied to the clinical assessment of neurological patients. The available information about the reliability of scores originating from this test version is even more limited in comparison to the other WCST versions. The M-WCST manual (Schretlen, 2010) provides a sole reliability estimate of .50 for its main composite measure of executive function, which combines number of categories and perseverative errors. This reliability coefficient was derived from a subsample of the standardization sample for which 5.5-year test-retest measurements were available (*n* = 103; without further specification of sample characteristics). A reliability of .50 would clearly discourage the clinical application of any test due to its intolerable susceptibility to measurement error. One of the main aims of the present study was to reexamine the reliability of W/M/M-W//CST scores. More specifically, we supposed that the rather disappointing reliability estimates that have been obtained by Lineweaver et al. (1999), Schretlen (2010), and other researchers might have originated from particular characteristics of the previous studies.

In classical test theory, reliability is closely linked to the notion of measurement error (Spearman, 1904). Equation 1 illustrates that the reliability of a measure decreases with proportional increases in error variance,

(1) $$rel_{xx}=\frac{\sigma_{x}^{2}-\sigma_{e}^{2}}{\sigma_{x}^{2}}=1-\frac{\sigma_{e}^{2}}{\sigma_{x}^{2}}$$

However, Equation 1 also reveals that reliability is a function of the variance of the people being assessed (i.e., $\sigma_{x}^{2}$) for fixed amount of error variance (i.e., $\sigma_{e}^{2}$). For example, if $\sigma_{e}^{2}$ = 10 and $\sigma_{x}^{2}$ = 20 then $rel_{xx}$ = .50, whereas if $\sigma_{e}^{2}$ = 10 and $\sigma_{x}^{2}$ = 100 then $rel_{xx}$ = .90. As a general rule, increasing interindividual variance without increasing the error variance will increase reliability, but decreasing interindividual variance without decreasing the error variance will decrease reliability. Hence, reliability emerges as a joint property of the test (i.e., its susceptibility to error) and the people being measured by the test (i.e., their variability on the measure of interest). In other words, the critical point here is that reliability estimates characterize the scores produced by a test in a particular setting, not the test itself. This issue remains poorly investigated though it has been frequently reiterated in the psychometric literature (Caruso, 2000; Feldt & Brennan, 1989; Wilkinson, 1999; Yin & Fan, 2000).

As outlined above, the same test may demonstrate different reliabilities in different contexts, and reliability generalization needs to be demonstrated rather being presumed (Cooper, Gonthier, Barch, & Braver, 2017; Thompson & Vacha-Haase, 2000). It is a widely held misconception that reliability coefficients from previous samples or from test manuals are applicable for the current context. Vacha-Haase, Kogan, and Thompson (2000) referred to this process as reliability induction. Empirical studies showed that reliability inductions are frequently inappropriate (Vacha-Haase et al., 2000; Whittington, 1998). Pedhazur and Schmelkin (1991) noted, “Such information may be useful for comparative purposes, but it is imperative to recognize that the relevant reliability estimate is the one obtained for the sample used in the study under consideration” (p. 86). It is this almost always unknown coefficient that governs the reliability of a measure, not the coefficient from previous samples or from test manuals. These considerations stimulate the question of whether the inadequate estimate of M-WCST reliability that was obtained from a non-clinical sample (Schretlen, 2010) generalizes to clinical settings. Often, the variability of a clinical sample grossly diverges from the variability of the normative sample from which the reliability coefficients are inducted (Crocker & Algina, 1986, p. 144): Reliability estimates from non-clinical samples might underestimate the reliability of M-WCST as it is applied in clinical practice due to, for example, the lower variability of the measures of interest in non-clinical compared to clinical samples. To test this possibility, we analyzed M-WCST data from 146 neurological inpatients referred for neuropsychological assessment. Against the background of the reliability estimate from a non-clinical sample, this clientele allows to study the generalizability of M-WCST reliability estimates.

In addition, we addressed a second limitation of the available W/M/M-W//CST reliability studies, that is, their sole reliance on test-retest reliability. Estimates of the reliability of M-WCST measures were obtained in the exemplary studies by Lineweaver et al. (1999) and Schretlen (2010) by correlating data obtained from two distinct measurements that were separated by long-lasting time intervals (Kopp, Maldonado et al., 2019, also Appendix A Table S1 in the Supplemental Appendix available online). Reliability estimates that are based on such test-retest data specifically pertain to the stability of the measures under consideration. For many clinical applications, other facets of reliability (e.g., internal consistency) seem more appropriate and informative, and it is surprising that these facets of reliability remained completely unexplored to date with regard to the W/M/M-W//CST. Estimates of consistency reliability are of indispensable importance for the computation of standard errors of measurement, estimated true scores, and hence, for calculating appropriate confidence intervals (McManus, 2012; Slick, 2006). Supplemental Appendix B (available online) describes fictitious examples, in which we highlight the impact that consistency reliability exerts on clinical decision making. In addition, some W/M/M-W//CST-specific characteristics might affect test-retest correlations while being less relevant for the test’s internal consistency (e.g., the requirement to establish the three cognitive sets for the first time; test demands may change drastically once they are established). To enrich the knowledge about the psychometric properties of the M-WCST, we conducted a fine-grained analysis of the split-half reliability of its major outcome measures.

Split-half reliability allows for estimating reliability whenever indices of performance on the task are collected from repeatedly administered trials. This requirement is clearly met by many psychological tasks that are routinely administered in experimental psychology and in neuropsychology (e.g., Cooper et al., 2017; Green et al., 2016; Hedge, Powell, & Sumner, 2018; Hilsabeck, 2017; Kowalczyk & Grange, 2017; Miller & Ulrich, 2013; Parsons, Kruijt, & Fox, 2019; Rouder & Haaf, 2019). Split-half reliability offers the unsurpassable advantage over test-retest reliability that there is no need to administer the task at hand repeatedly over time to the identical sample of people. Rather than that, a single administration of the task is sufficient to estimate how reliable the measured indices of task performance are in the particular sample under study. Note also that trial-level measures of many types, such as dichotomy (e.g., failure/success on each trial), polychotomy (e.g., a categorical scale on each trial), and continuity (e.g., response times on each trial) are eligible for the calculation of split-half reliability because it rests on adding single-trial outcomes together across subsets of trials.

The M-WCST, for example, can be divided into two discretionary construed test halves (i.e., the composites first and second test half, respectively), rendering it possible to calculate indices of task performance (e.g., by adding up all perseverative errors) separately for each of the two test halves. Split-half reliability estimates reflect the degree of their congruence, which is quantified by calculating their correlation, corrected for test length (see Method section for details). In addition to this split between the first and the second test half, composites (i.e., test halves) can also be formed based on an odd/even trial-number split, for which adjacent trials are assigned to distinct test halves.

In general, split-half reliability estimates from odd/even-splits will surpass those from first/second half-splits, mainly for two reasons: First, transient environmental events (take distraction by a task-irrelevant stimulus as a conceivable example) have a stronger detrimental impact on first/second half-splits (e.g., distraction might have been present during the first test half but absent during the second test half) than on odd/even splits (short-lived distraction has the potential to exert its influence on (nearly) as many odd as on even trials). Second, interindividual variability with regard to long-term trends (such as learning or fatigue as easily conceivable examples) exerts a stronger detrimental impact on first/second half-splits (e.g., one subject may show better performance on the second test half due to learning how to handle the task at hand; another subject may show worse performance on the second test half due to the preponderance of fatigue) than on odd/even splits, which typically are more resistant to individual differences in non-stationary trends.

The existence of different options to split tests into composites leaves selecting “its” split-half reliability at the discretion of the researcher, who may choose between reporting relatively high odd/even-coefficients and typically lower first/second coefficients (Lord, 1956). Here, we utilized sampling-based iteration techniques (Berry, Johnston, & Mielke, 2011; Berry, Johnston, Mielke, & Johnston, 2018) to obtain unbiased estimates of split-half reliability. Building on random test splits allows overcoming the potential biases toward higher or lower ends of the potential range of reliability coefficients, which are associated with discretionary construed test splits (e.g., odd/even trial numbers or first/second test halves). Rather than that, sampling-based methods allow to obtain representative estimates of split-half reliabilities (MacLeod et al., 2010; Parsons, 2017; Pauly, Umlauft, & Ünlü, 2018; Revelle, 2018; Sherman, 2015).

In sum, we provide the first estimates of split-half reliability of M-WCST scores, based on a sample drawn from the population of patients to which the W/M/M-W//CST versions are typically administered. To obtain estimates of split-half reliability of M-WCST scores, we calculated reliabilities from discretionary construed test splits, ranging from very short-grained splits (i.e., odd/even trial numbers) to very long-grained splits (i.e., first/second test halves). In addition, we also calculated estimates of split-half reliability by building random M-WCST splits.

## Method

### Data Collection

We analyzed data that were obtained from 146 (58 female; *M* = 61.69 years; *SD* = 12.35 years) inpatients who were consecutively referred by handling neurologists for a neuropsychological evaluation by an experienced neuropsychologist (BK). The study received institutional ethics approval (Ethikkommission at the Hannover Medical School; Vote 7529) and was in accordance with the 1964 Helsinki declaration and its later amendments. Written informed consent was obtained from participants. The patients were referred for the presence of a variety of suspected neurological conditions. The final diagnoses that the patients received were frontotemporal lobar degeneration (*n* = 26), atypical Parkinson’s disease (progressive supranuclear palsy, cortico-basal degeneration, multisystem atrophy–Parkinsonian subtype; *n* = 25), Alzheimer’s disease/mild cognitive impairment (*n* = 18), multiple sclerosis (*n* = 14), normal pressure hydrocephalus (*n* = 14), depression (*n* = 13), stroke (*n* = 13), neuropathy (*n* = 10), vascular encephalopathy (*n* = 10), and no neurological (or psychiatric) disease (*n* = 3). We report sociodemographic and neuropsychological characteristics as well as task performance of the diagnostic subgroups in a related paper (Kopp, Steinke, Bertram, Skripuletz, & Lange, 2019). All assessments were conducted in a standardized, calm environment that precluded distraction by caregivers, relatives, or patients.

Note that this sample already represents a selection of patients from the referral group for which the administration of the M-WCST seemed to be indicated as part of their assessment. The main exclusion criteria were intellectual disability, advanced dementia or severe depression that would preclude successful performance on the M-WCST. The exclusion of patients was based on clinical judgment by the neuropsychologist who was responsible for the study (BK). There were no quantitative cutoffs applied to the selection decisions. Patients who showed clinical signs of any of these conditions were transferred to assessment procedures that seem to be more adequate for these clienteles because an administration of the relatively difficult M-WCST might have been harmful to these patients.

Most—but not all—of the 146 patients who were selected for M-WCST assessment were completing at least major parts of the M-WCST, as described below in detail. For an analysis of the complete M-WCST, we excluded all patients who completed less than the maximum of 48 trials (with a range of 3 to 28 trials; *n* = 18; 4 female; *M* = 64.94 years; *SD* = 14.52 years). Exclusion of those patients resulted in a final sample of *n* = 128 patients (88% of the originally preselected group of 146 patients; 54 female; *M* = 61.23 years; *SD* = 12.01 years) for that purpose. For an analysis of only the first test half, we excluded all patients who completed less than the initial 50% (i.e., 24) of the M-WCST trials (with a range of 3 to 17 trials; *n* = 9; 1 female; *M* = 66.22 years; *SD* = 9.11 years). Exclusion of those patients resulted in a final sample of *n* = 137 (94% of the originally preselected group of 146 patients; 57 female; *M* = 61.39 years; *SD* = 12.50 years) for that purpose.

### Materials and Design

All included patients underwent administration of the M-WCST for neuropsychological assessment. Our reliability analysis comprises four basic indices of performance on the task: First, the number of correct sorts (*n_corr*) is simply an overall count of successful sorts (i.e., trials which resulted in positive feedback). Second, the number of categories (*n_cat*) is an overall count of the categories achieved. Following Nelson (1976), the M-WCST manual (Schretlen, 2010) asks individuals to produce only six consecutive successful sorts to complete a category. To render the number of categories suitable for split-half reliability analyses, we assigned a score of 1/6 to each of the six trials constituting a run of consecutive successful sorts (i.e., a category). For example, a patient who achieved three categories would receive a score of 1/6 on 18 trials (i.e., three times six consecutive successful sorts), resulting in an overall *n_cat* = 18 * 1/6 = 3. Of importance, this procedural detail allowed calculating split-half reliability estimates of *n_cat*, simply because each individual trial could be assigned to one of the two test halves. For example, if 10 of these trials were included in the category score on one test half and 8 trials on the other half, the test halves would gain category scores of *n_cat_1_* = 10 * 1/6 = 1.67 and *n_cat_2_* = 8 * 1/6 = 1.33, respectively. Third, the number of perseverative errors (*n_PE*) equals the number of failures to shift cognitive set in response to negative feedback. We also counted the number of trials on which a perseverative error could occur (*n_poss_PE*), but this variable was not subjected to reliability analyses. Fourth, the number of set-loss errors (*n_SE*) equals the number of failures to maintain cognitive set in response to positive feedback. We also counted the number of trials on which a set-loss error could occur (*n_poss_SE*), but this variable was again not subjected to reliability analyses.

In addition to the four basic M-WCST scores (i.e., *n_corr, n_cat, n_PE, n_SE*), 11 linear combinations of interest were calculated from these basic scores. Those 11 composite indices were [*n_PE* + *n_SE, n_corr* + *n_cat, n_corr* + *n_PE, n_corr* + *n_SE, n_corr* + *n_PE* + *n_SE, n_cat* + *n_PE, n_cat* + *n_SE, n_cat* + *n_PE* + *n_SE, n_corr* + *n_cat* + *n_PE, n_corr* + *n_cat* + *n_SE*, and *n_corr* + *n_cat* + *n_PE* + *n_SE*]. For this purpose, the basic scores were standardized using the *z* transformation,

(2) $$z(x_{jk})=\frac{x_{jk}-M_{k}}{SD_{k}}.$$

For patient *j* on a defined set of trials *k* (i.e., a particular test half), the *z* score $z(x_{jk})$ was computed as the individual score $x_{jk}$ minus the sample mean $M_{k}$, divided by the sample standard deviation $SD_{k}$. Note that the mean and standard deviation were based on the scores obtained from all patients on a set of trials *k*. In addition, we multiplied the number of perseverative and set-loss errors by minus one, as these scores were inverted to the number of correct sorts and categories. This reversal of the sign was required because the number of perseverative and set-loss errors and the number of correct sorts and categories have opposite signs in their correlations with task performance (e.g., fewer perseverative errors are signs of better task performance, but fewer categories indicate worse task performance). For example, the linear combination of number of categories and number of perseverative errors for patient *j* on set *k* was calculated as

(3) $${\left(n\_cat+n\_PE\right)}_{jk}=z\left(n\_cat_{jk}\right)-z\left(n\_PE_{jk}\right).$$

The linear combination of number of categories and perseverative errors approximates the *Executive Function Composite* as described in the M-WCST manual (Schretlen, 2010), whereas the linear combination of perseverative and set-loss errors is similar to the often utilized count of the total number of errors. Table 1 illustrates that most of these linear combinations resulted in internally consistent composite measures. Coefficients *alpha* were lowest when number of set-loss errors formed part of the composite. For example, the internal homogeneity of the two scores that summarized failures (i.e., number of perseverative errors and set-loss errors) should be considered as being insufficient (α = .344 for the complete test, α = .007 for the initial 24 trials) for combining them to a single indicator of task performance.

**Table 1.** Coefficients Alpha (α) for Various Subsets of M-WCST Scores That Were Obtained From the Administration of the Complete Test (48 Trials) or the First Test Half (24 Initial Trials).

	Complete test (48 trials; *N* = 128)	First test half (24 trials; *N* = 137)
*n_PE* / *n_SE*	.344	.007
*n_corr* / *n_cat*	.937	.876
*n_corr* / *n_PE*	.952	.939
*n_corr* / *n_SE*	.519	.196
*n_corr* / *n_PE* / *n_SE*	.742	.599
*n_cat* / *n_PE*	.873	.814
*n_cat* / *n_SE*	.806	.673
*n_cat* / *n_PE* / *n_SE*	.787	.665
*n_corr* / *n_cat* / *n_PE*	.947	.916
*n_corr* / *n_cat* / *n_SE*	.840	.723
*n_corr* / *n_cat* / *n_PE* / *n_SE*	.873	.797

*Note*. M-WCST = Modified Wisconsin Card Sorting Test; *n_PE* = number of perseverative errors; *n_SE* = number of set-loss errors; *n_corr* = number of correct sorts; *n_cat* = number of categories.

### Statistical Analysis

**Figure 1.**
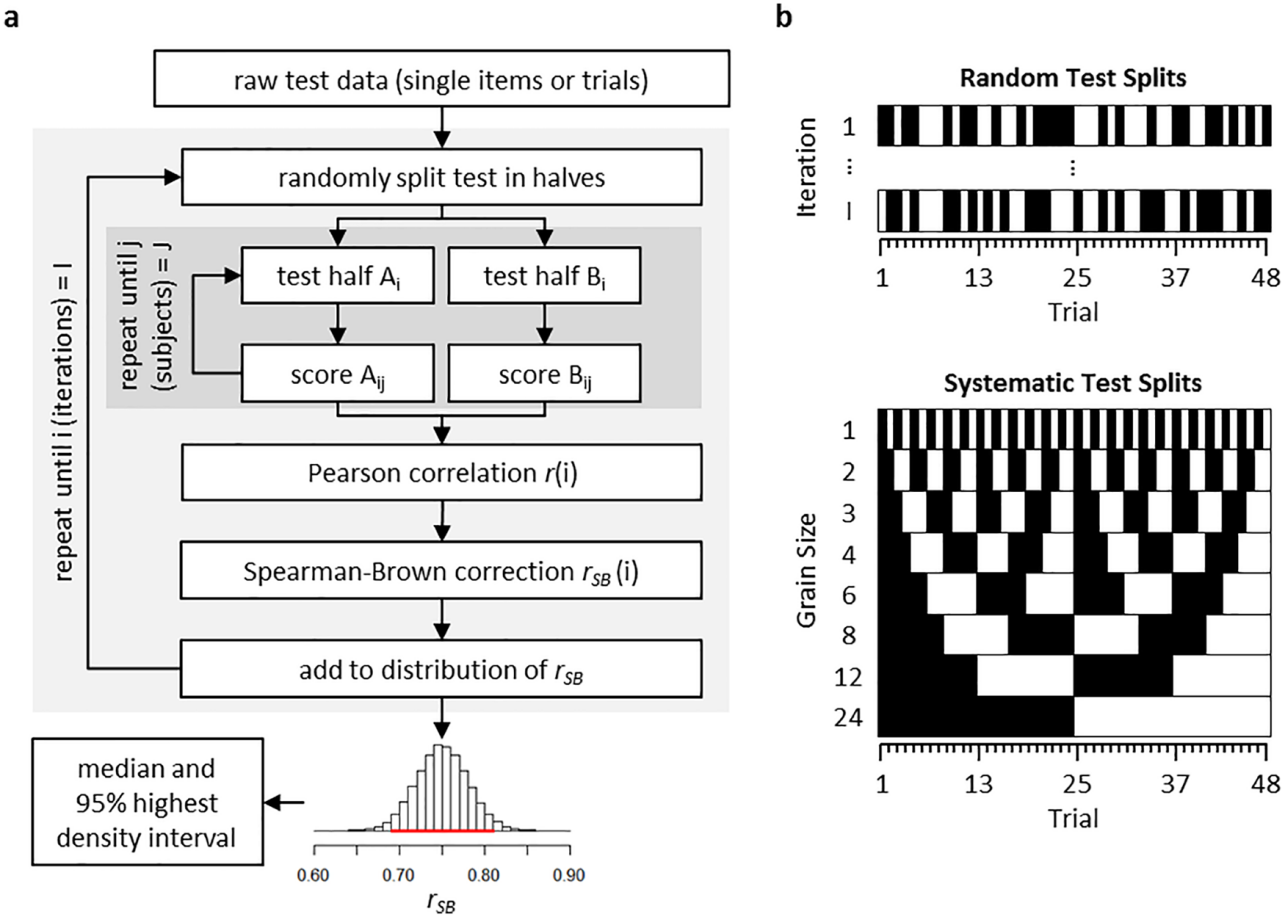
(a) An outline of our sampling approach for estimating split-half reliabilities. The procedure is appropriate for all kinds of data obtained from tests that are composed of multiple items or repeated trials. On any iteration *i*, the overall set of items or trials was split into two halves of equal length, *A_i_* and *B_i_*, respectively. For each patient *j* with *j* = (1, . . ., *J*), the test score of interest was calculated separately for the two test halves, yielding two scores, *A_ij_* and *B_ij_* for each individual. The Pearson correlation coefficient *r*(*i*) was calculated across all *A_ij_* and *B_ij_* pairings, which was finally corrected for test length by the well-known Spearman–Brown formula (*r_SB_*(*i*); Brown, 1910; Spearman, 1910). The emergent *r_SB_*(*i*) was stored. Further inferences were based on the distribution of all *r_SB_*(*i*) for I = 100,000 iterations. The median and the 95% highest density intervals (*HDI*; shown as horizontal lines in red color in the histograms) of the emergent distribution were computed as estimates of the central tendency and uncertainty of split-half reliability estimates, respectively. (b) For the purpose of our reliability analysis, we considered random and systematic splits of scores that were derived from test halves. Exemplary test halves are shown in black and white for illustration. Random test splits such as the exemplary one shown here were used for the sampling approach (as described in detail in Part (a) of this figure). Systematic test splits included different ways to split the test into halves. With 48 trials, a relatively large number of systematic splits can be construed that differ with regard to their “grain size.” The odd/even split (with a grain size of 1 trial) and the first/second test half split (with a grain size of 24 trials) are most commonly utilized for that purpose. In addition to these two splits, we also divided the test into systematic halves based on grain sizes of 2, 3, 4, 6, 8, and 12 consecutive trials. The emergent reliability estimates are reported.

Figure 1 illustrates how we estimated split-half reliabilities for the four basic and eleven combined M-WCST scores. Pearson correlations r of test scores that were obtained on two test halves were computed as an estimate of reliability (Figure 1A). However, the resulting correlation coefficient *r* needed correction for test length via the well-known Spearman–Brown formula (Brown, 1910; Spearman, 1910),

(4) $$r_{SB}=\frac{2r}{1+r}.$$

Figure 1a also shows how we utilized sampling-based iteration techniques to obtain representative estimates of split-half reliability across all 48 trials of the M-WCST. Sampling-based methods for the estimation of split-half reliability had been proposed in the form of freely available R packages (*splithalf*: Parsons, 2017; *psych*: Revelle, 2018; *multicon*: Sherman, 2015; see also MacLeod et al., 2010). To that end, reliability estimates were repeatedly computed for random test splits, resulting in a sampling-based distribution of reliability estimates. We report the median of the distribution of these sampled $r_{SB}$ as representative estimates of split-half reliabilities. The uncertainty associated with these central-tendency estimates was quantified by the 95% highest density interval (*HDI*) of the distribution. The 95% *HDI* gives the interval for $r_{SB}$ that contains 95% of the split-half reliabilities that were obtained from the random sampling of test splits. The median and the 95% *HDI* were reported as robust summary statistics, because we could not make any assumptions about the shapes of reliability distributions. The here reported split-half reliability estimates were based on 100,000 iterations each.

In addition to the sampling-based approach, we also performed test splits in a systematic manner, as shown in Figure 1b. To that end, we manipulated the grain size of the test halves, from the shortest possible grain size (one trial, constituting the so-called odd-even split) to the longest possible grain size (24 trials, constituting the so-called first-second halves split). Note that the latter method can be considered as estimating test-retest reliabilities at the shortest possible time intervals, since the cards in the two sets of 24 response cards appear in exactly identical serial orderings. Thus, the serial order of response cards on trial 25 to 48 is an exact replication of the serial order of response cards on trial 1 to 24. Furthermore, we also analyzed all intermediate grain sizes that could be created on 48 trials. First, the odd/even split (i.e., trials 1, 3, 5, . . ., 47 vs. trials 2, 4, 6, . . ., 46, 48) represents a grain size of one trial, with the shortest possible average lag of only one single trial (i.e., trial 2 minus trial 1, . . ., trial 48 minus trial 47). Second, a grain size of two trials (i.e., trials 1, 2, 5, 6, . . ., 46 vs. trials 3, 4, . . ., 47, 48) is also associated with an average lag of two trials (i.e., trial 3 minus trial 1, . . ., trial 48 minus trial 46). These splitting procedures were also exercised for grain sizes 3, 4, 6, 8, 12, and finally 24 trials (i.e., test halves; trials 1-24 vs. trials 25-48), in which case the longest possible average lag of 24 trials emerged (i.e., trial 25 minus trial 1, . . ., trial 48 minus trial 24).

We excluded the following grain sizes from the systematic analyses for some of the measurements: First, grain sizes of one and three trials were not eligible for analysis of the number of categories (*n_cat*) and its combinations with any other basic measure (*n_corr, n_PE, n_SE*). This was due to the fact that with six consecutive successful sorts to complete a category, their trial-wise count (as explained above) would be equally distributed across the two test halves with grain sizes of either one or three trials. Hence, a seeming reliability of 1 would emerge under these conditions. Second, a grain size of one trial was not eligible for analysis of the number of set-loss errors (*n_SE*) and its combinations with any other basic measure (*n_corr, n_cat, n_PE*). This was due to the fact that when a set-loss error occurred on trial *t −* 1, the occurrence of an additional set-loss error on the next trial *t* was impossible by definition because the feedback on trial *t −* 1 would have been negative. Hence, a seeming lack of reliability would emerge under these conditions.

All reliability analyses were conducted for the complete test (i.e., 48 trials) and the first test half only (i.e., the first 24 trials). Analyses of the first test half were conducted to see how reliability estimates were affected by a drastic shortening of test length. Information from solely the first test half might be of importance because some patients failed to complete the entire series of 48 trials on the M-WCST (in the sample that we report here, 50% of the patients who failed to complete 48 trials [*n* = 18] completed at least 24 trials [*n* = 9]). Note that splitting trials into grains of 8 and 24 consecutive trials was impossible when analyzing the first test half (Trials 1 to 24). All analyses were conducted using R Version 3.4.2 (R Core Team, 2017).

## Results

### Descriptive Statistics of M-WCST Scores

Table 2 shows the results from descriptive analyses of the data obtained from those *n* = 128 patients who completed all 48 M-WCST trials. The number of correct sorts amounted to an average of little more than 24 trials, indicating that merely around 50% of all sorts were successes. Patients achieved slightly less than three categories and committed more than nine perseverative errors on average. These perseverative errors were committed on an average of around 23 trials that offered the opportunity to commit such an error, indicating that the conditional probability for the occurrence of perseverative errors amounted to .41. At the same time, patients committed less than three set-loss errors on average. These set-loss errors were committed on an average of around 24 trials that offered to opportunity to commit such an error (conditional probability for the occurrence of set-loss errors of .12). Inspection of Table 3 reveals that the data obtained (from the first test half only) of those *n* = 137 patients who completed not less than 24 M-WCST trials showed very similar trends.

**Table 2.** Descriptive Statistics of M-WCST Scores, *M* (*SD*), on the Complete Test (*N* = 128).

		Odd/even trials		First/second test halves	
	All trials (1, 2, . . ., 48)	Odd trials (1, 3, . . ., 47)	Even trials (2, 4, . . ., 48)	First (trial 1-24)	Second (trial 25-48)
*n_corr*	24.38 (9.76)	12.07 (4.91)	12.31 (5.01)	12.85 (5.15)	11.53 (6.02)
*n_cat*	2.95 (1.88)	1.47 (0.94)	1.47 (0.94)	1.63 (1.00)	1.31 (1.07)
*n_PE*	9.39 (8.53)	4.59 (4.26)	4.80 (4.47)	4.06 (4.46)	5.33 (4.99)
*n_SE*	2.84 (2.83)	1.45 (1.63)	1.40 (1.83)	1.32 (1.53)	1.52 (1.79)
*n_poss_PE*	23.09 (9.51)	11.16 (4.75)	11.93 (4.91)	10.66 (4.90)	12.43 (5.91)
*n_poss_SE*	23.91 (9.51)	11.84 (4.75)	12.07 (4.91)	12.34 (4.90)	11.57 (5.91)

*Note*. M-WCST = Modified Wisconsin Card Sorting Test; *n_corr* = number of correct sorts; *n_cat* = number of categories; *n_PE* = number of perseveration errors; *n_SE* = number of set-loss errors; *n_poss_PE* = number of perseveration errors possible; *n_poss_SE* = number of set-loss errors possible.

**Table 3.** Descriptive Statistics of M-WCST Scores, *M* (*SD*), on the First Test Half (*N* = 137).

		Odd/even trials		First/Second Test Halves	
	All trials (1, 2, . . ., 24)	Odd trials (1, 3, . . ., 23)	Even trials (2,4, . . ., 24)	First (trial 1-12)	Second (trial 13-24)
*n_corr*	12.25 (5.60)	6.08 (2.86)	6.17 (2.88)	6.14 (3.13)	6.11 (3.50)
*n_cat*	1.42 (0.98)	0.71 (0.49)	0.71 (0.49)	0.77 (0.57)	0.65 (0.61)
*n_PE*	4.67 (5.09)	2.20 (2.51)	2.47 (2.73)	2.19 (2.69)	2.48 (2.85)
*n_SE*	1.28 (1.50)	0.55 (0.90)	0.72 (1.10)	0.54 (0.80)	0.74 (1.01)
*n_poss_PE*	11.23 (5.34)	5.31 (2.62)	5.92 (2.86)	5.42 (2.85)	5.81 (3.47)
*n_poss_SE*	11.77 (5.34)	5.69 (2.62)	6.08 (2.86)	5.58 (2.85)	6.19 (3.47)

*Note*. M-WCST = Modified Wisconsin Card Sorting Test; *n_corr* = number of correct sorts; *n_cat* = number of categories; *n_PE* = number of perseveration errors; *n_SE* = number of set-loss errors; *n_poss_PE* = number of perseveration errors possible; *n_poss_SE* = number of set-loss errors possible.

### Split-Half Reliability

**Figure 2.**
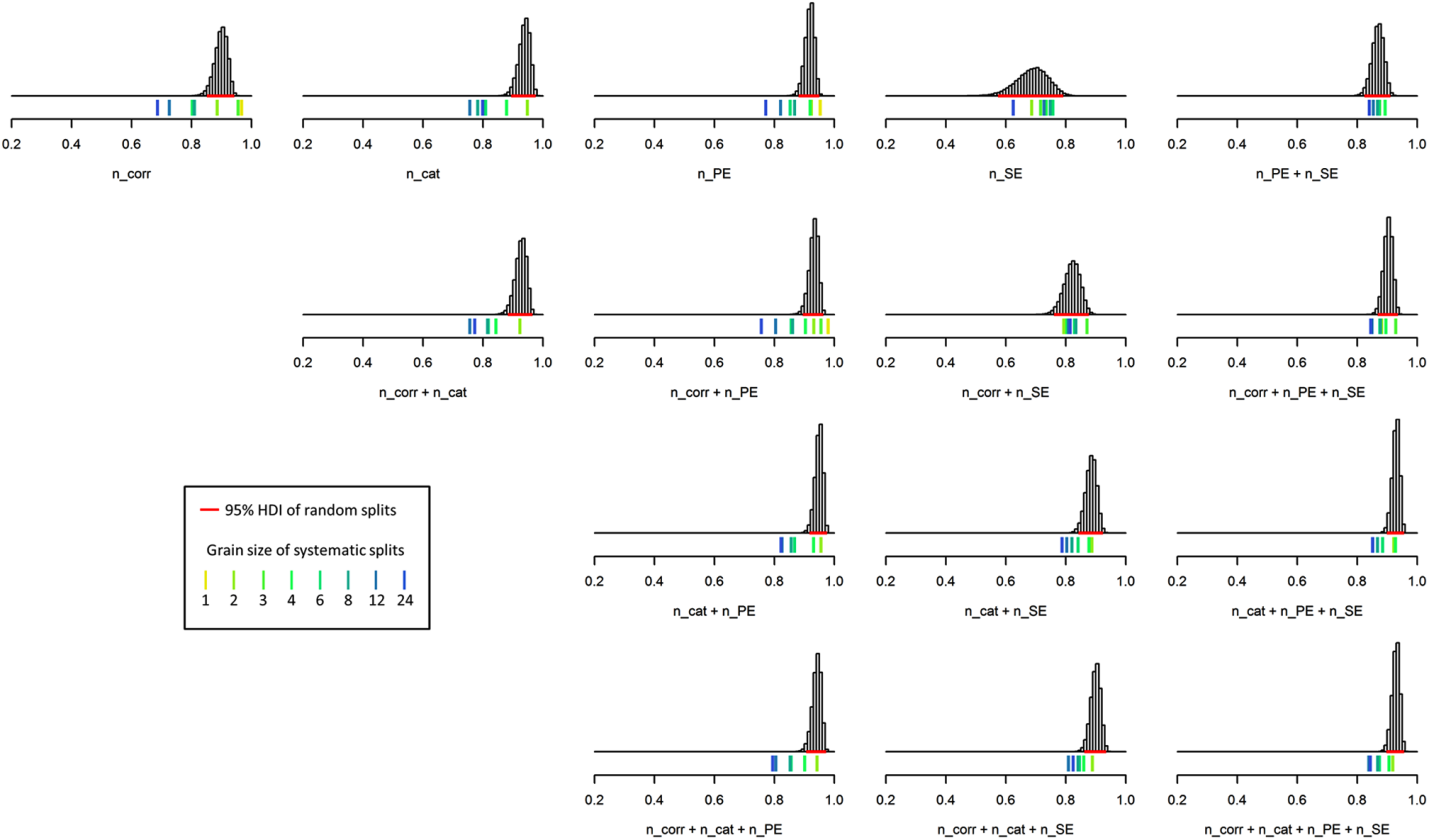
Distribution of reliability estimates on the complete test (48 trials). *Note. n_corr* = number of correct sorts; *n_cat* = number of categories; *n_PE* = number of perseverative errors; *n_SE* = number of set-loss errors. Histograms show the frequency of split-half reliability estimates obtained from 100,000 random splits (95% highest density intervals [*HDI*s] shown as horizontal lines in red color). Colored ticks indicate the Spearman–Brown coefficients that were obtained from systematic splits, with the yellow-to-blue dimension indicating the split’s grain size.

Table 4 and Figure 2 show the results from reliability analyses of the data obtained from those *n* = 128 patients who completed all 48 M-WCST trials. The randomly sampled splits revealed that the number of categories was the most reliable basic indicator of task performance, followed by the number of perseverative errors and the number of correct sorts. The three split-half reliability estimates fell in the range between .90 ≤ *r_SB_* ≤ .95, whereas the number of set-loss errors was clearly less reliable (*r_SB_* < .70) than the other three basic measures. Two observations from randomly sampled splits for the composite indicators of task performance appear noteworthy. First, those composite indices that include the number of set-loss errors as the sole measure of failures are somewhat lower (*r_SB_* ≤ .90) than all other composite indices (*r_SB_* ≥ .93). Second, the maximal reliability estimate was achieved by combining number of categories and perseverative errors (*r_SB_* = .95), which approximates the *Executive Function Composite* of the M-WCST (Schretlen, 2010).

**Table 4.** Spearman–Brown Split-Half Reliability Coefficients (*r_SB_*) for M-WCST Scores (and Linear Combinations Thereof) on the Complete Test (48 Trials; *N* = 128).

	Systematic splits (trial grain size)								Random splits		
										95% *HDI*	
	1	2	3	4	6	8	12	24	*Mdn*	Low	High
*n_corr*	.967	.886	.956	.802	.806	.810	.726	.687	.900	.856	.938
*n_cat*	na	.948	na	.879	.809	.782	.756	.799	.939	.899	.971
*n_PE*	.953	.923	.919	.919	.853	.868	.820	.772	.919	.886	.946
*n_SE*	na	.687	.716	.735	.757	.748	.727	.625	.691	.579	.787
*n_PE* + *n_SE*	na	.841	.875	.893	.873	.867	.854	.840	.870	.829	.906
*n_corr* + *n_cat*	na	.923	na	.844	.818	.816	.756	.773	.928	.888	.961
*n_corr* + *n_PE*	.979	.932	.955	.904	.856	.861	.804	.756	.933	.900	.959
*n_corr* + *n_SE*	na	.794	.871	.803	.829	.834	.809	.815	.822	.766	.872
*n_corr* + *n_PE* + *n_SE*	na	.881	.928	.896	.876	.877	.849	.846	.904	.873	.931
*n_cat* + *n_PE*	na	.956	na	.931	.867	.856	.826	.822	.950	.922	.972
*n_cat* + *n_SE*	na	.888	na	.877	.842	.821	.804	.787	.885	.847	.919
*n_cat* + *n_PE* + *n_SE*	na	.922	na	.927	.885	.868	.852	.851	.930	.904	.951
*n_corr* + *n_cat* + *n_PE*	na	.943	na	.902	.856	.853	.805	.794	.943	.912	.968
*n_corr* + *n_cat* + *n_SE*	na	.889	na	.860	.846	.841	.809	.824	.900	.867	.930
*n_corr* + *n_cat* + *n_PE* + *n_SE*	na	.919	na	.906	.874	.868	.838	.843	.930	.902	.952

*Note*. M-WCST = Modified Wisconsin Card Sorting Test; *HDI* = highest density interval (low = lower limit at 95% credibility; high = higher limit at 95% credibility); *n_corr* = number of correct sorts; *n_cat* = number of categories; *n_PE* = number of perseverative errors; *n_SE* = number of set-loss errors; na = not applicable. *Median* and *HDI* of Spearman–Brown coefficients from 100,000 random splits per cell are reported.

The eight systematic splits revealed that reliabilities were—as expected—negatively correlated with grain size. For example, the reliability estimate for the number of perseverative errors fell more or less continuously from *r_SB_* = .953 on odd/even-splits (grain size = 1) to *r_SB_* = .772 on first/second-half-splits (grain size = 24). The number of set-loss errors (either alone or in combination) constituted the sole exception from this rule, with all reliability estimates for the number of set-loss errors lying close to .65, *r_SB_* = .687 (grain size = 2) and *r_SB_* = .625 (grain size = 24).

**Figure 3.**
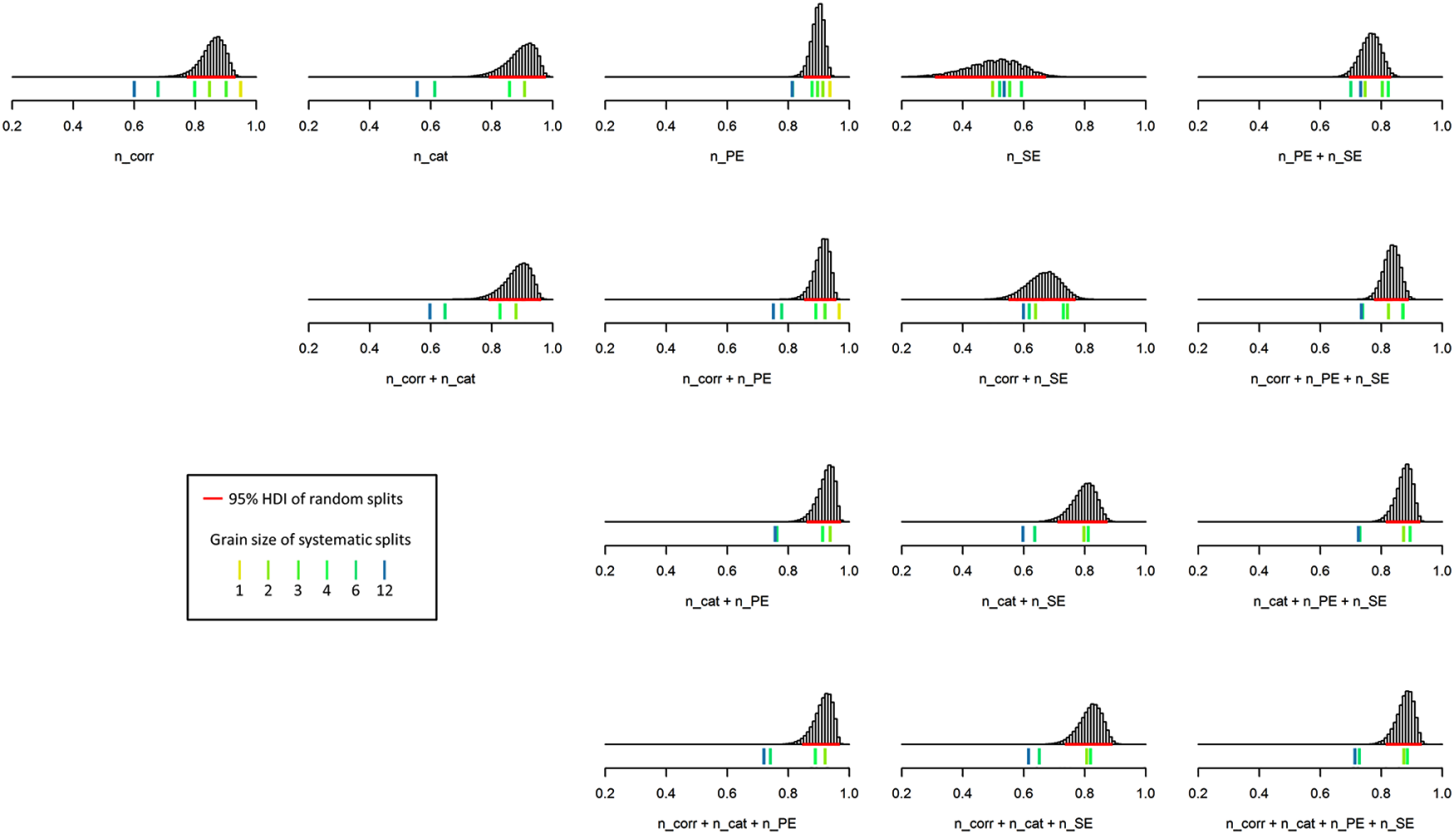
Distribution of reliability estimates on the first test half (initial 24 trials). *Note. n_corr* = number of correct sorts; *n_cat* = number of categories; *n_PE* = number of perseverative errors; *n_SE* = number of set-loss errors. Histograms show the frequency of split-half reliability estimates obtained from 100,000 random splits (95% highest density intervals [*HDI*s] shown as horizontal lines in red color). Colored ticks indicate the Spearman–Brown coefficients that were obtained from systematic splits, with the yellow-to-blue dimension indicating the split’s grain size.

Table 5 and Figure 3 show the results from reliability analyses of the data obtained from those *n*=137 patients who completed not less than 24 M-WCST trials. Similar to what we observed from the analysis of the complete test, the number of categories appeared to be most reliable, the number of set-loss errors stood out as the least reliable basic measure. Again, combining number of categories and perseverative errors yielded the most reliable composite index, and reliability estimates generally decreased with increasing grain size.

**Table 5.** Spearman–Brown Split-Half Reliability Coefficients (*r_SB_*) for M-WCST Scores (and Linear Combinations Thereof) on the First Test Half (24 Trials; *N* = 137).

		Systematic splits (trial grain size)					Random splits		
	1	2	3	4	6	12	*Mdn*	95% *HDI*	
								low	high
*n_corr*	.949	.847	.901	.799	.678	.600	.862	.777	.928
*n_cat*	na	.908	na	.859	.613	.556	.903	.795	.973
*n_PE*	.937	.913	.896	.878	.814	.813	.898	.855	.935
*n_SE*	na	.498	.554	.592	.521	.536	.509	.314	.668
*n_PE* + *n_SE*	na	.747	.803	.823	.701	.732	.767	.699	.828
*n_corr* + *n_cat*	na	.879	na	.827	.647	.598	.890	.794	.957
*n_corr* + *n_PE*	.967	.920	.921	.890	.779	.752	.913	.857	.953
*n_corr* + *n_SE*	na	.639	.744	.730	.618	.599	.668	.555	.765
*n_corr* + *n_PE* + *n_SE*	na	.824	.872	.871	.739	.735	.835	.781	.886
*n_cat* + *n_PE*	na	.938	na	.913	.763	.757	.927	.865	.969
*n_cat* + *n_SE*	na	.798	na	.812	.636	.598	.802	.715	.870
*n_cat* + *n_PE* + *n_SE*	na	.873	na	.895	.730	.725	.878	.820	.923
*n_corr* + *n_cat* + *n_PE*	na	.921	na	.889	.741	.720	.918	.850	.965
*n_corr* + *n_cat* + *n_SE*	na	.807	na	.819	.651	.616	.822	.740	.887
*n_corr* + *n_cat* + *n_PE* + *n_SE*	na	.874	na	.886	.728	.714	.881	.820	.929

*Note*. M-WCST = Modified Wisconsin Card Sorting Test; *HDI* = highest density interval (low = lower limit at 95% credibility; high = higher limit at 95% credibility); *n_corr* = number of correct sorts; *n_cat* = number of categories; *n_PE* = number of perseverative errors; *n_SE* = number of set-loss errors; na = not applicable. *Median* and *HDI* of Spearman–Brown reliability coefficients from 100,000 random splits per cell are reported.

## Discussion

We outlined in the Introduction that reliability relates to the sample from which it is estimated and cannot be immediately generalized. Our study provides an example for non-generalizability of reliability because M-WCST measures obtained from neurological patients showed good reliabilities, which contrasts with the bulk of the published studies on WCST reliability that were predominantly obtained from non-clinical samples. Our data clearly establish the legitimacy of the M-WCST (Schretlen, 2010) as a reliable neuropsychological tool for the assessment of executive function *in neurological patients*. In particular, they reveal that the sampling-based split-half reliability of the *Executive Function Composite* (i.e., the combination of number of categories and perseverative errors) equals .95, within 95% credibility limits between .922 and .972. The main conclusion from this finding is that the reliability of this measure can be considered as being satisfactorily assured under the assumption of its administration in the context of the clinical assessment of neurological patients. At this point, it should be recalled that influential psychometricians regarded a reliability of .95 as the desirable standard (Nunnally & Bernstein, 1994). Our results terminate the practice of the more or less blind-flying application of the M-WCST in clinical neuropsychology. They allow practitioners administering this test of eminent importance with peace of conscience, despite the lack of confidence induced by most of the published reliability estimates that were available to date (Kopp, Steinke, et al., 2019).

However, the non-generalizability of reliability renders it still necessary to estimate reliability within single studies. According to the most recent standards for psychological testing (American Psychological Association, American Educational Research Association, & National Council on Measurement in Education, 2014), indices of reliability refer to measures obtained in particular samples that the studies were examining, rather than to the measures in general. Hence, reliability cannot be considered as a property of a measure that would be invariant across samples. An integral part of all behavioral sciences therefore includes reporting reliability estimates for the considered measures, whenever possible, from the clinician’s or researcher’s sample or samples (Appelbaum et al., 2018). In that regard, it is of importance that our study also delivers a general method for quantifying sample-wise split-half reliability estimates. In that regard, we implemented our method in an easily applicable, Excel-based software tool with a graphical user interface, which is suitable for the evaluation of sample-wise reliability estimates (Steinke & Kopp, 2019).

Among other surprisingly favorable findings, we report here that the split-half reliability of the *Executive Function Composite* seems to satisfy the desirable standard of .95. The M-WCST manual (Schretlen, 2010) itself estimated the test-retest reliability of this composite measure as being merely .50, thereby putting the legitimacy of the M-WCST as a means to quantitatively assess abilities in executive function into doubt. The two reliability estimates are irreconcilable because the estimate of .50 lies far below the lower limit of 95% *HDI*, which in case of the *Executive Function Composite* amounted to .922 in our study. Two disparities in the way these two reliabilities were estimated seem to be important for understanding their divergence: First, we analyzed the reliability of the *Executive Function Composite* in a patient sample, whereas the manual’s estimate was based on a subsample of the standardization sample. Second, we estimated the *Executive Function Composite* split-half reliability, whereas the reliability coefficient that was reported in the manual estimated test-retest reliability originating from a very long (5.5 years) time interval between the two test administrations. Practitioners who utilize the M-WCST for the purpose of clinical assessment are advised to rely on our results when considering the psychometric quality of the emergent measures: They are typically concerned with a clientele that bears more similarities with our sample of patients than with the manual’s sample of people. In addition, they are usually not so much interested in predicting future performance on the M-WCST than in the internal consistency of their measures.

One should keep in mind Equation 1 when comparing reliability estimates that were obtained from samples of patients and those obtained from non-patient samples: At (roughly) fixed error variance (reflecting a measure’s precision), higher amounts of interindividual variance maximize reliability, whereas lower amounts of interindividual variance minimize reliability, with the consequence that the same test may demonstrate different reliabilities in different contexts (Caruso, 2000; Cooper et al., 2017; Feldt & Brennan, 1989; Wilkinson, 1999; Yin & Fan, 2000). Per definition, neurological alterations induce variability on neuropsychological test performance. It is thus not surprising to observe substantial interindividual variation in M-WCST performance in our sample of neurological patients. For example, at an average number of *M (n_PE)* > 9 occurrences, perseverative errors occurred with a standard deviation of *SD (n_PE)* > 8. Values for both indicators of task performance (average, variability) are likely to be much lower in high-functioning non-patient samples, which can partly account for the usually lower reliability estimates obtained from these samples. Further illustrating this principle, we found substantially lower reliability estimates within our sample for a performance measure with less interindividual variability (i.e., the number of set-loss errors, *M(n_SE)* < 3, *SD(n_SE)* < 3). To conclude, the degree of fit between subject characteristics needs to be considered when one tries to generalize reliability estimates that had been reported in psychometric studies to the circumstances of test administration under scrutiny (Crocker & Algina, 1986).

The other issue derives from discrepancies between test-retest reliability and split-half reliability. Test-retest reliability provides an estimate of the correlation between two measures from the same test administered at two different points in time to identical participants (Slick, 2006). All measures have infinite numbers of possible test-retest reliabilities, depending on the length of the time interval between test administrations. While measures with good temporal stability show little change over time, measures of less stable abilities produce decreasing test-retest reliabilities with increasing time intervals. As we have shown here, systematic variations of grain size for estimates of split-half reliability may be considered as serving the same purpose. Specifically, increasing grain size successively converts split-half reliability from a measure of internal consistency, that is, the consistency of composites such as test halves, to a measure of consistency over time, that is, to a measure of temporal stability. Our data revealed that many M-WCST measures (the number of correct sorts, categories, and perseverative errors) displayed decreasing split-half reliabilities with increasing grain sizes, pointing into the direction of limited temporal stability of these measures. It comes therefore as no surprise that estimates of test-retest reliability of these measures fall below corresponding split-half estimates. As noted by one of the reviewers, the issues that we discussed here are to a certain extent beyond our current knowledge. We included the paragraph in question because we wanted to offer a potential explanation—open to discussion—for the observed negative correlations between grain size and the resulting reliability coefficients.

The many possible ways to compute split-half reliabilities offer two different ways in its interpretation as a test’s reliability: First, split-half reliability as an indicator of internal consistency of composites may be best assessable via odd/even test splits at minimum grain size. Split-half reliabilities resulting from test splits at maximum grain size (i.e., first/second test halves) should in contrast be preferably considered as estimations of temporal stability. Although the emergent estimates can vary for different choices of grain size, their discrepancy does not necessarily indicate a contradiction. Researchers can simply choose whether they want reliability estimates of internal consistency of composites or of short-term temporal stability. Researchers should solely communicate the goals associated with estimating a test’s split-half reliability as clearly as possible. Second, the sampling-based method of estimating split-half reliability offers the opportunity to compromise in an elegant manner between the named extremes (Lord, 1956; MacLeod et al., 2010; Parsons, 2017; Pauly et al., 2018; Revelle, 2018; Sherman, 2015). The application of this method yields unbiased estimates of split-half reliability, which is based on large numbers of iteratively sampled random grain sizes. Random sampling also offers the advantage that its application provides credibility intervals rather than point estimates, thereby guarding against chance when comparing reliabilities.

There are also practical reasons for choosing split-half reliability because split-half reliability estimates rest on one single administration of a test to a sample of people. For example, our split-half reliability estimates rested on one single administration of the M-WCST to a sample of patients. We would like to highlight the potential to divide nearly all psychological tests into composites (halves) and to estimate reliabilities by way of analyzing the relationships between the measures emerging from these splits. The published literature on W/MCST reliability reveals that this avenue did not yet receive sufficient appreciation by the field, which rather relied on test-retest reliability approaches. This approach presupposes that tests were administered twice to an identical sample of people, thus rendering the acquisition of appropriate data difficult and costly. Consequent to this laborious acquisition of relevant data, many of the relatively few published reliability studies comprised insufficient sample sizes (see Kopp, Maldonado, et al., 2019, see also Appendix A Table S1 in the Supplemental Appendix, available online). Computing split-half reliabilities offers the potential to circumvent this detrimental bottleneck of clinical neuropsychology in the future. In fact, each practitioner can utilize his or her data sets for the named purpose. Solely trial-level data are required that were obtained from a test administered once to a sample of people. In case of the M-WCST, a digitized version of the handwritten record tables obtained from a sample of people constitutes a sufficient data base for conducting such analyses. A boost in the number of reliability studies could rapidly enhance our psychometric knowledge about neuropsychological tests, thereby strengthening the psychometric foundation of neuropsychology.

Such a strengthening is badly required because evaluating measures in terms of their reliability remains an indispensable building block of any psychological assessment. Slick (2006) provided an easily comprehensible introduction that summarized how a measure’s reliability should influence practitioners with regard to the degree of uncertainty that remains associated with the test scores they observe. Without going into details here, it suffices to remind us that the estimated true score, the standard errors of estimation and measurement, and the confidence interval around the measured scores strongly depend on that measure’s reliability (Charter, 2003; Crawford, 2012; Slick, 2006). In diagnostic practice, however, reliability is often neglected. This happens in part because test manuals often do not provide sufficient and/or appropriate information about a measure’s reliability nor guidance for computing the reliability-referenced quantities that were mentioned above. For example, the M-WCST manual (Schretlen, 2010) asserts that “adequate” reliability estimates exist, while actually ignoring the measure’s reliabilities during the diagnostic process.

Heaton’s standard WCST version (Heaton et al., 1993) utilized as much as 128 trials (there is also 64-trial version of the standard version, Kongs, Thompson, Iverson, & Heaton, 2000), and Nelson’s (1976) MCST version as well as Schretlen’s (2010) M-WCST version get along with 48 trials. Our results imply that even 24 trials might render sufficiently reliable M-WCST measures, provided that it is utilized for the clinical assessment of neurological patients for whom long-during psychological testing may constitute substantial distress, in particular under conditions of prevalent failure. Notably, the sampling-based split-half reliability of the *Executive Function Composite* (i.e., the composite of the number of categories and perseverative errors) equaled .927 when it was estimated solely from the initial 24 trials, within 95% credibility limits between .865 and .969. This finding indicates that the *Executive Function Composite* seems to possess good split-half reliability, even when recorded from a considerably abbreviated test version.

### Limitations and Suggestions

With regard to the robustness of reliability estimates, sample size is one of the main limiting factors (Charter, 1999). While being comparable to the largest sample size available in previous reliability studies (Lineweaver et al., 1999), our study’s sample size still might have been too small to obtain precise point estimates of split-half reliability. However, one of the advantages of the applied sampling-based method is that it does not only yield (necessarily imperfect) point estimates of reliability but also a quantification of the imprecision associated with these estimates (i.e., credibility intervals). Many reliability estimates reported here show overlapping credibility intervals, limiting our confidence in the significance of differences between them. For example, the sampling-based estimate for the number of perseverative errors amounted to .919, with a 95% credibility interval of .886 to .946. The composite measure comprising the number of perseverative errors and set-loss errors achieved the maximal reliability estimate, .95, but clearly the lower limit of the 95% credibility interval, .922, falls within the credibility interval that was associated with the number of perseverative errors. The assurance of the significance of small differences in reliability requires larger sample sizes, which might be achievable through the application of meta-analytic methods (Botella, Suero, & Gambara, 2010; Feldt & Charter, 2006; Henson & Thompson, 2002; Howell & Shields, 2008; López-Pina et al., 2015; Mason, Allam, & Brannick, 2007; Sánchez-Meca, López-López, & López-Pina, 2013; Vacha-Haase, 1998).

It is also worth noticing that we administered the M-WCST in a manner that deviated in certain aspects from the specifications in the test manual (Schretlen, 2010). There were three modifications: First, the succession of task rules had to be carried out in fixed order, with the specific sequence {color, shape, number, color, shape, number}. This modification follows the standard WCST version (Heaton et al., 1993). Second, rule switches were not announced verbally. The introduction of a verbal announcement of rule switches dates back to Nelson’s (1976) MCST version. However, we are convinced that verbal announcement of rules switches changes the nature of the original task in an unfavorable direction. Again, our modification follows the standard WCST version (Heaton et al., 1993). Third, we utilized slightly modified task instructions compared to those documented in the M-WCST manual. Our wording was developed over two decades of clinical experience with the administration of the WCST to neurological patients, and it is documented in Supplemental Appendix C (available online). We do not expect that these differences in M-WCST administration exert noticeable effects on the psychometric characteristics of the test’s measures, although they may limit the generalizability of reliability estimates from our study to studies that employ the original M-WCST version. However, an important corollary of our work is that reliability estimates will be different in each studied sample, calling for studies on reliability generalization.

The three modifications that were made in our administration of the M-WCST are inconsistent with the administration of the M-WCST as published by Schretlen (2010). As noted by one of the reviewers, the results may show intact reliability, but do not speak to the validity of the test scores. Further research related to validity and not just reliability alone is needed in order to understand the full psychometric properties of the M-WCST administered according to our modifications.

### Conclusions

Reliability remains the primary psychometric property of neuropsychological measures, and split-half reliability offers to many researchers and practitioners manifold opportunities to evaluate the reliability of the measures they are relying on, based on a single administration of a test to a sample of people. A boost in the number of reliability studies is badly required because the available knowledge about the psychometric quality of neuropsychological assessment is currently unsatisfactory. In particular, the systematic investigation of reliability generalization was up to date severely neglected, forcing practitioners into blind-sighted application of neuropsychological tests. The results reported here are very encouraging because they suggest that the WCST—which represents the most eminent test of executive function— yields reliable measures for clinical assessment, despite less promising previous data concerning that matter. The apparent discrepancy between the present and previous findings seems to follow a comprehensible logic, the two main issues being the characteristics of the studied sample and the specific indicator of reliability reported. Based on the considerations discussed above, we recommend that split-half reliability be preferentially reported in future psychometric studies in as many patient samples as possible.

## Supplemental Material

supplement_material – Supplemental material for The Reliability of the Wisconsin Card Sorting Test in Clinical Practice Click here for additional data file. Supplemental material, supplement_material for The Reliability of the Wisconsin Card Sorting Test in Clinical Practice by Bruno Kopp, Florian Lange and Alexander Steinke in Assessment
