# Supplementary material for: The Reliability of the Wisconsin Card Sorting Test in Clinical Practice
Source: Assessment. 2019 Aug 2;28(1):248–63. doi: 10.1177/1073191119866257 (PMC7780274; doi:10.1177/1073191119866257)
Supplement: supplement_material – Supplemental material for The Reliability of the Wisconsin Card Sorting Test in Clinical Practice [file supplement_material.pdf]

The reliability of the Wisconsin Card Sorting Test in clinical practice

- Appendix -

APPENDIX A

Reliability estimates for common WCST measures

The available reliability studies of common WCST measures are summarized in Table S1. It is worth noting that our compilation of reliability studies does not pretend being exhaustive because our search for suitable studies was not systematic. Having said this, inspection of Table S1 nonetheless reveals that estimates of WCST internal consistency remained completely unavailable.

All studies relied on repeated administration of the WCST, estimating test-retest-reliabilities with variable retest periods. Nine studies examined the administration of a WCST version in non-clinical populations (cumulative *N* = 568), implying that the five studies of clinical populations achieved a cumulative *N* of only 120 patients. In addition, the diagnoses under consideration were quite heterogeneous (traumatic brain injury, sleep apnoe syndrome, autism, learning disability). Very little is known about test-retest reliability when the WCST is administered in clinical populations that are of major interest for neuropsychologists. In that regard, one has to rely on the estimates from those two studies that looked at patients who suffered from traumatic brain injury (cumulative *N* = 57; Greve, Bianchini, Mathias, Houston, & Crouch, 2002; Tate, Perdices, & Maggiotto, 1998). However, the results from these two studies can hardly be considered as being convergent. In addition, many different coefficients were considered across these studies (Pearson's *r*, Spearman's *rho*, Kendall's *tau*, intra-class correlations, generalizability coefficients).

Table S1

A summary of published studies on W/MCST reliability.

Study	Year	Population	*N*	Type of Reliability	Test-Retest Interval	WCST Version	WCST Measure	Coefficient	Type	Test *M* (*SD*) or *Med (Range)**	Retest *M* (*SD*) or *Med (Range)**
**Basso et al.**	1999	Non-clinical	50	Test-retest	12 months	Heaton et al. 1993	CAT	.54	6	5.16 (1.38)	5.42 (1.55)
							TE	.50	6	26.12 (18.04)	16.68 (11.88)
							PE	.52	6	14.20 (10.53)	8.44 (6.16)
							P	.50	6	16.02 (12.82)	9.34 (7.70)
							FMS	-.02	6	1.16 (1.67)	0.80 (1.16)
**Bird et al.**	2004	Non-clinical	90	Test-retest	1 month	Nelson 1976	TE	.34	1	5.0 (0-22)*	3.0 (0-19)*
							PE	.38	1	1.0 (0-9)*	0.0 (0-7)*
**Bowden et al.**	1998	Non-clinical	75	Test-retest ('alternate' forms)	Same day	Heaton et al. 1981	CAT	.60	2	5.35 (1.35)	5.6 (0.85)
							TE	.51	2	28.95 (19.2)	24.5 (12.0)
							PE	.32	2	15.2 (12.1)	7.55 (6.1)
							P	.30	2	17.5 (14.7)	11.8 (8.6)
							NPE	.43	2	13.85 (8.95)	9.45 (8.95)
**de Zubicaray et al.**	1998	Non-clinical	36	Test-retest	7.5 months	Nelson 1976	CAT	.28	2	6 (1-6)*	6 (1-6)*
							TE	.36	2	8.5 (1-39)*	7.5 (1-26)*
							PE	.27	2	2.5 (0-18)*	2.0 (0-13)*
							NPE	.38	2	6.0 (1-32)*	6.0 (0-44)*
							FMS	.49	2	0 (0-6)*	0 (0-4)*
**Greve et al.**	2002	TBI	34	Test-retest	66 weeks	Heaton et al. 1993	CAT	.53	3	2.97 (2.49)	3.91 (2.33)
							TE	.82	2	52.47 (26.88)	43.24 (25.07)
							PE	.80	2	34.35 (13.89)	25.85 (17.23)
							P	78	2	42.47 (34.29)	30.32 (27.41)
							NPE	.50	2	17.74 (11.30)	16.94 (9.56)
							FMS	.26	3	1.47 (1.71)	1.44 (1.37)
**Heaton et al.**	1993	Non-clinical	46	Test-retest	33 days	Heaton et al. 1993	TE	.71	5	n.a.	n.a.
							PE, P	.52,.53	5	n.a.	n.a.
							NPE	.72	5	n.a.	n.a.
**Ingram et al.**	1999	Sleep apnoe patients	29	Test-retest	12 days	Computerized WCST	CAT	.70	2	n.a.	n.a.
							TE	.79	2	n.a.	n.a.
							PE	.83	2	n.a.	n.a.
							P	.79		n.a.	n.a.
							FMS	.50	2	n.a.	n.a.
**Lineweaver et al.**	1999	Non-clinical	142	Test-retest	24 months	Nelson 1976	CAT	.56	1	5.09 (1.43)**	n.a.
							PE	.64	1	2.32 (5.02)**	n.a.
							NPE	.46	1	7.92 (6.51)**	n.a.
**Ozonoff**	1995	Autistic children	17	Test-retest	30 months	Standard WCST	TE	.94	5	n.a.	n.a.
							P	.93	5	n.a.	n.a.
		Learning disabled children and adolescents	17	Test- retest	30 months	Standard WCST	TE	.90	5	n.a.	n.a.
							P	.94	5	n.a.	n.a.
**Paolo et al.**	1996	Non-clinical	87	Test-retest	12 months	Heaton et al. 1981	CAT	.65	3	4.84 (1.76)	4.86 (1.89)
							TE	.66	3	n.a.	n.a.
							PE	.65	3	n.a.	n.a.
							P	.63	3	n.a.	n.a.
							NPE	.55	3	n.a.	n.a.
							FMS	.13	3	1.16 (1.39)	0.69 (1.20)
**Steinmetz** et al.	2010	Non-clinical	22	Test-retest	Same day	Heaton et al. 1993	TE	.68	6	n.a.	n.a.
							PE	.72	6	n.a.	n.a.
							FMS	.16	6	n.a.	n.a.
Tate et al.	1998	Non-clinical	20	Test-retest	8 months	Heaton et al. 1993	CAT	.88	2	5.05 (1.36)	5.25 (1.62)
							TE	.79	4	28.2 (20.2)	24.25 (22.91)
							PE	.72	4	14.1 (10.84)	10.95 (8.42)
							P	.68	4	15.55 (12.21)	11.9 (10.03)
							NPE	.74	4	14.1 (13.25)	13.3 (16.61)
							FMS	-.04	4	1.35 (1.53)	1.05 (1.28)
		TBI	23	Test-retest	10 months	Heaton et al. 1993	CAT	.29	2	4.0 (1.54)	5.13 (1.79)
							TE	.39	4	45.91 (21.78)	29.3 (23.74)
							PE	.34	4	30.52 (20.02)	16.04 (13.4)
							P	.33	4	37.35 (26.39)	18.04 (16.19)
							NPE	.32	4	15.39 (8.16)	13.22 (11.38)
							FMS	-.32	4	0.96 (1.22)	0.61 (0.78)

Note. CAT = categories; TE = total errors; PE, P = perseveration errors, perseverations; NPE = non-perseverative errors; FMS = failures to maintain set; TBI = traumatic brain injury; 1 = Pearson's *r*, 2 = Spearman's *rho*, 3 = Kendall's *tau*, 4 = intra-class coefficient, 5 = generalizability coefficient, 6 = unspecified; **Lineweaver (N = 229).

APPENDIX B

Consistency reliability and clinical decision making

McManus (2012) provides a practical overview of interpretative problems that are related to the standard error of measurement (*SEM*). There are three different *SEMs* (Dudek, 1979), which McManus (2012) refers to as *SEmeas*, *SEest*, and *SEpred*, in order to avoid confusion. Assume that we measured two identical standard scores (z with M = 0; SD = 1) from an examinee (z = -1.50), score z_A from an assessment instrument with relatively low consistency reliability (rel_A = .6), and score z_B from a more reliable assessment instrument (rel_B = .9).

The standard error of measurement, *SEmeas*, provides an estimate of the variability of the actual scores given (unknown) true scores, with confidence intervals (CIs) that are symmetric around measured scores. *SEmeas* is useful as a general measure for comparing assessment instruments. According to Equation B1,

$$SEmeas = SD\sqrt{1 - rel} \qquad \text{(Eq. B1).}$$

$SEmeas_A$ equals .63, and the $CI(A)_{95\%}$ = -1.50±1.96*.63 = -2.74…-0.26, such that $CI(A)_{95\%}$ excludes 0. $SEmeas_B$ equals .32, and the $\mathrm{CI(B)}_{95\%}$ = -1.50±1.96*.32 = -2.12…-0.88, such that $CI(B)_{95\%}$ excludes 0. Thus, both CIs exclude zero, leading the examiner to conclude that both actual scores fall below zero.

SEest estimates the variability of the true score, given the measured score. It is this quantity that the diagnostician should mainly be interested in. Importantly, one has to take regression to the mean into account, with the consequence that CIs are asymmetric around measured scores (and may not even include the measured score). Estimated true scores (ETS) are calculated according to Equation B2,

$$ETS = M + rel\,(obs - M) \qquad \text{(Eq. B2).}$$

Hence ETS_A equals -0.90 and ETS_B equals -1.35. According to Equation B3,

$$SEest = SD\sqrt{rel(1 - rel)} \qquad \text{(Eq. B3).}$$

$SEest_A$ equals .49, and the $CI(A)_{95\%}$ = -0.90$\pm$1.96*.49 = -1.86…+0.06, such that $CI(A)_{95\%}$ includes 0. $SEest_B$ equals .30; and the $CI(B)_{95\%}$ = -1.35$\pm$1.96*.30 = -1.94…-0.76, such that $CI(B)_{95\%}$ excludes 0. Thus, the examiner would conclude that the true score on A (with low reliability) cannot be distinguished from zero, whereas the identical observed score on B (with higher reliability) leads the examiner to conclude that the true score on B falls below zero.

SEpred estimates the variability of future observed scores from measured scores. Again, regression to the mean needs to be taken into account. According to Equation B4,

$$SEpred = SD\sqrt{1 - rel^2} \quad \text{(Eq. B4).}$$

$SEpred_A$ equals .80, and the $CI(A)_{95\%}$ = -0.90$\pm$1.96*.80 = -2.47…+0.67, such that $CI(A)_{95\%}$ includes 0. $SEpred_B$ equals =.44, and the $CI(B)_{95\%}$ = -1.35$\pm$1.96*.44 = -2.20…-0.50, such that $CI(B)_{95\%}$ excludes 0. As in the example above, the examiner would reach opposite conclusions under these circumstances.

APPENDIX C

Documentation of task instructions

“You see four stimulus cards in front of you. Before we start, I want you to inspect each single stimulus card and to think about what these cards depict.

In addition to the four stimulus cars, I have a deck of response cards that you will receive in a minute. Your task, then, is to match each of the response cards, one after the other, to one of the four stimulus cards.

That is, *(here I use the first response card [comment, i.e. four red crosses, these words are not part of the instruction], and I demonstrate that it could be matched to the stimulus card depicting four blue circles, or three yellow crosses, OR one red triangle)*. You will decide where you want to place your response card, BUT there is a matching rule, which renders only one of your choices correct, whereas the other potential choices will be incorrect.

The problem here is that I cannot inform you about the correct matching rule. It is your task to find out this correct matching rule. How can you achieve this? You achieve it by listening to the feedback that I will provide after each sort. That is, I will tell you after every sort whether the response card has its ‘correct’ position (the cards match according to the rule) or ‘incorrect’ position (the card do not match according to the rule).

Here I give you an example: If you place your response card *(the one that depicts four red crosses)* here *(stimulus card = two green stars)*, my answer would probably be ‘incorrect’. In case of an ‘incorrect’-feedback, we will not correct the position of the incorrectly placed response card. You will just pick up the next response card, and you will try to match that response card correctly with one of the stimulus cards.

Before we start, here is one additional important piece of information for you. The correct matching rule will change from time to time. I will not inform you when these rule changes will happen, but you will recognize their occurrence through changes in my feedback behavior. This means, previously correct sorts would then be incorrect sorts.

Let’s go!”

References

Basso, M. R., Bornstein, R. A., & Lang, J. M. (1999). Practice effects on commonly used measures of executive function across twelve months. *The Clinical Neuropsychologist*, *13*(3), 283–292. https://doi.org/10.1076/clin.13.3.283.1743

Bird, C. M., Papadopoulou, K., Ricciardelli, P., Rossor, M. N., & Cipolotti, L. (2004). Monitoring cognitive changes: Psychometric properties of six cognitive tests. *British Journal of Clinical Psychology*, *43*(2), 197–210. https://doi.org/10.1348/014466504323088051

Bowden, S. C., Fowler, K. S., Bell, R. C., Whelan, G., Clifford, C. C., Ritter, A. J., & Long, C. M. (1998). The reliability and internal validity of the Wisconsin Card Sorting Test. *Neuropsychological Rehabilitation*, *8*(3), 243–254. https://doi.org/10.1080/713755573

de Zubicaray, G. I., Smith, G. A., Chalk, J. B., & Semple, J. (1998). The Modified Card Sorting Test: Test-retest stability and relationships with demographic variables in a healthy older adult sample. *British Journal of Clinical Psychology*, *37*(4), 457–466. https://doi.org/10.1111/j.2044-8260.1998.tb01403.x

Dudek, F. J. (1979). The continuing misinterpretation of the standard error of measurement. *Psychological Bulletin*, *86*(2), 335–337. https://doi.org/10.1037/0033-2909.86.2.335

Greve, K. W., Bianchini, K. J., Mathias, C. W., Houston, R. J., & Crouch, J. A. (2002). Detecting malingered performance with the Wisconsin Card Sorting Test: A preliminary investigation in traumatic brain injury. *The Clinical Neuropsychologist*, *16*(2), 179–191. https://doi.org/10.1076/clin.16.2.179.13241

Heaton, R. K., Chelune, G. J., Talley, J. L., Kay, G. G., & Curtiss, G. (1993). *Wisconsin Card Sorting Test (WCST) manual: Revised and expanded*. Odessa, FL: Psychological Assessment Resources.

Ingram, F., Greve, K. W., Fishel Ingram, P. T., & Soukup, V. M. (1999). Temporal stability of the Wisconsin Card Sorting Test in an untreated patient sample. *British Journal of Clinical Psychology*, *38*(2), 209–211. https://doi.org/10.1348/014466599162764

Lineweaver, T. T., Bondi, M. W., Thomas, R. G., & Salmon, D. P. (1999). A normative study of Nelson's

(1976) modified version of the Wisconsin Card Sorting Test in healthy older adults. *The Clinical Neuropsychologist*, *13*(3), 328–347. https://doi.org/10.1076/clin.13.3.328.1745

McManus, I. C. (2012). The misinterpretation of the standard error of measurement in medical education: A primer on the problems, pitfalls and peculiarities of the three different standard errors of measurement. *Medical Teacher*, *34*(7), 569–576. https://doi.org/10.3109/0142159X.2012.670318

Ozonoff, S. (1995). Reliability and validity of the Wisconsin Card Sorting Test in studies of autism. *Neuropsychology*, *9*(4), 491–500. https://doi.org/10.1037/0894-4105.9.4.491

Paolo, A. M., Axelrod, B. N., & Tröster, A. I. (1996). Test-retest stability of the Wisconsin Card Sorting Test. *Assessment*, *3*(2), 137–143. https://doi.org/10.1177/107319119600300205

Steinmetz, J.-P., Brunner, M., Loarer, E., & Houssemand, C. (2010). Incomplete psychometric equivalence of scores obtained on the manual and the computer version of the Wisconsin Card Sorting Test? *Psychological Assessment*, *22*(1), 199–202. https://doi.org/10.1037/a0017661

Tate, R. L., Perdices, M., & Maggiotto, S. (1998). Stability of the Wisconsin Card Sorting Test and the determination of reliability of change in scores. *The Clinical Neuropsychologist*, *12*(3), 348–357. https://doi.org/10.1076/clin.12.3.348.1988
